# Structural and Functional Analysis of Human HtrA3 Protease and Its Subdomains

**DOI:** 10.1371/journal.pone.0131142

**Published:** 2015-06-25

**Authors:** Przemyslaw Glaza, Jerzy Osipiuk, Tomasz Wenta, Dorota Zurawa-Janicka, Miroslaw Jarzab, Adam Lesner, Bogdan Banecki, Joanna Skorko-Glonek, Andrzej Joachimiak, Barbara Lipinska

**Affiliations:** 1 Department of Biochemistry, Faculty of Biology, University of Gdansk, 80–308 Gdansk, Poland; 2 Midwest Center for Structural Genomics, Argonne National Laboratory, Argonne, Illinois, IL 60439, United States of America; 3 Structural Biology Center, Biosciences Division, Argonne National Laboratory, Argonne, Illinois, IL 60439, United States of America; 4 Department of Biochemistry, Faculty of Chemistry, University of Gdansk, 80–308 Gdansk, Poland; 5 Department of Molecular and Cellular Biology, Intercollegiate Faculty of Biotechnology of the University of Gdansk and the Medical University of Gdansk, 80–822 Gdansk, Poland; Centro Nacional de Biotecnologia - CSIC, SPAIN

## Abstract

Human HtrA3 protease, which induces mitochondria-mediated apoptosis, can be a tumor suppressor and a potential therapeutic target in the treatment of cancer. However, there is little information about its structure and biochemical properties. HtrA3 is composed of an N-terminal domain not required for proteolytic activity, a central serine protease domain and a C-terminal PDZ domain. HtrA3S, its short natural isoform, lacks the PDZ domain which is substituted by a stretch of 7 C-terminal amino acid residues, unique for this isoform. This paper presents the crystal structure of the HtrA3 protease domain together with the PDZ domain (ΔN-HtrA3), showing that the protein forms a trimer whose protease domains are similar to those of human HtrA1 and HtrA2. The ΔN-HtrA3 PDZ domains are placed in a position intermediate between that in the flat saucer-like HtrA1 SAXS structure and the compact pyramidal HtrA2 X-ray structure. The PDZ domain interacts closely with the LB loop of the protease domain in a way not found in other human HtrAs. ΔN-HtrA3 with the PDZ removed (ΔN-HtrA3-ΔPDZ) and an N-terminally truncated HtrA3S (ΔN-HtrA3S) were fully active at a wide range of temperatures and their substrate affinity was not impaired. This indicates that the PDZ domain is dispensable for HtrA3 activity. As determined by size exclusion chromatography, ΔN-HtrA3 formed stable trimers while both ΔN-HtrA3-ΔPDZ and ΔN-HtrA3S were monomeric. This suggests that the presence of the PDZ domain, unlike in HtrA1 and HtrA2, influences HtrA3 trimer formation. The unique C-terminal sequence of ΔN-HtrA3S appeared to have little effect on activity and oligomerization. Additionally, we examined the cleavage specificity of ΔN-HtrA3. Results reported in this paper provide new insights into the structure and function of ΔN-HtrA3, which seems to have a unique combination of features among human HtrA proteases.

## Introduction

Human HtrA3, a proapoptotic protein, is a member of the HtrA (high temperature requirement A) family of homo-oligomeric serine proteases, which are well conserved in evolution and whose primary function is to maintain protein quality control. Their common structural features are the presence of the chymotrypsin-like protease domain (PD) and at least one PDZ (post-synaptic density 95, *Drosophila* discs large, zona occludens-1) domain localized at the C-terminus. The N-terminus is variable and it may contain signal and regulatory sequences.

The common structural unit of the HtrA proteases is a pyramid-shaped trimer consisting of PDs, which form the central core, and outward-protruding PDZ domains. In some HtrAs, for example in the *Escherichia coli* HtrA(DegP) and DegQ, this trimeric unit may further oligomerize, forming hexamers, dodecamers, or icosatetramers [1] [2] [3]. Crystal structures of several HtrAs have been solved, including the *E*. *coli* HtrA(DegP) [1] [4], DegS [5] [6] and DegQ [2]; *Legionella falloni* DegQ [7]; *Mycobacterium tuberculosis* HtrA2 [8] and the human HtrA1 (PD and N-terminal domain) [9] [10], HtrA2 [11], and HtrA3 PDZ [12]. There are also two NMR structures of the HtrA1 PDZ domain available [12]. Substrate binding may lead to the formation of higher order oligomers and activation [3] [9] [13]. On the other hand, the loss of oligomeric structure may lead to inactivation, as in the case of human HtrA2 [11] [14].

PDZ domains have been shown to act as regulatory elements and as substrate specificity determinants [2] [15] [16] [17]. They bind the hydrophobic stretches of misfolded substrate (or regulatory) polypeptides, leading to structural changes in PDs and enzyme activation. In spite of the structural similarity of PDZ domains observed among HtrA proteases, their impact on activity varies: they may be indispensable for activity, as in the case of *E*. *coli* HtrA(DegP) PDZ1 [18], or dispensable, as in human HtrA1 [9]. The PDZ domains may also participate in oligomer formation (reviewed in [19]).

HtrA3 was initially identified in the developing placenta as a serine protease associated with pregnancy [20] [21] [22] [23]. Dysregulation of HtrA3 has been observed in a number of diseases including cancer [24] [25] [26] [27] [28] [29] [30] and preeclampsia [31] [32]. Downregulation of HtrA3 was associated with the progression of endometrial and ovarian cancer [26] [27] [28] [29]. HtrA3 downregulation in lung cancer results in resistance to chemotherapeutic treatments with etoposide and cisplatin. HtrA3 is involved in the induction of the intrinsic, mitochondria-mediated apoptotic pathway. Upon treatment with cytotoxic agents, HtrA3 is released from the mitochondrium into the cytosol, where it triggers apoptosis *via* its serine protease activity [24] [25]. HtrA3 is thus proposed to be a tumor suppressor and a potential therapeutic target in the cancer treatment [25] [33].

HtrA3 has two isoforms, long (HtrA3L) and short (HtrA3S), resulting from alternative mRNA splicing [21]. The long isoform of HtrA3 (HtrA3L) is a 49-kDa protein that contains a signal secretory peptide at the N-terminus (residues 1–17), a domain with homology to the insulin-like growth factor binding proteins (IGFBP) (residues 29–94) and a Kazal-type inhibitor motif (KI) (residues 89–126) followed by a serine protease domain (PD) with the catalytic triad H191-D227-S305 and one PDZ domain at the C-terminal end. In contrast to HtrA3L, the 39-kDa short isoform of HtrA3 (HtrA3S) lacks the PDZ domain. In place of the PDZ domain it has a stretch of seven amino acids (APSLAVH) at the C-terminus, which is unique for this isoform and is encoded by a separate exon [21]. The function of this structural element is not known. Both variants of HtrA3 are widely expressed in the human body with variable levels in different organs [21]. The HtrA3 proteins undergo autocleavage of the N-terminal region containing the IGFBP and KI motifs [24] [34] [35] [36]. The autocatalytic cleavage of the N-terminal domain from HtrA3L is necessary for mitochondrial to cytoplasmic translocation of the protease and increased cell death. Both the full length (residues 18–453) and N-terminally-truncated (residues ~ 130–453) forms of HtrA3L exist *in vivo* [25] [36]. The N-terminal domain is not required for the protease activity. Its deletion causes an effect similar to that observed for HtrA1 (~ 3-fold activity increase) [34]. Despite the physiological importance of HtrA3, little is known about its structure and function at the molecular level. Only the structure of the PDZ domain of HtrA3 has been determined by X-ray crystallography [12]. The oligomerization status of HtrA3 and the role of the PDZ domain for oligomer formation are still unknown. Also, the biochemical properties of HtrA3 are not well characterized.

In this paper we studied the N-terminally truncated form of HtrA3L, comprising the PD-PDZ region; here after referred to as ΔN-HtrA3. The purpose of the study was to solve the crystal structure of ΔN-HtrA3, assess the ΔN-HtrA3 oligomerization status in solution, and characterize its enzymatic activity, focusing on the role of the PDZ domain in proteolysis and structure formation. To complement these studies and to assess the role of the unique C-terminal stretch of seven amino acids of HtrA3S, the activity and oligomerization status of an N-terminally truncated HtrA3S (ΔN-HtrA3S) was also tested.

The crystal structure of recombinant HtrA3 purified from bacterial cells containing two domains, PD and PDZ (ΔN-HtrA3_S305A_, residues 130–453), at a resolution of 3.27 Å was determined. The S305A mutation eliminated the serine protease activity causing partial degradation of the ΔN-HtrA3 protein and thus assured a high level of homogeneity for preparations. The structure showed that ΔN-HtrA3 forms a trimer with PDs essentially similar to those of HtrA1 and HtrA2. We found that both ΔN-HtrA3-ΔPDZ and ΔN-HtrA3S were fully active compared to ΔN-HtrA3, indicating that the PDZ domain is dispensable for HtrA3 activity. As determined by size exclusion chromatography, ΔN-HtrA3 formed trimers while ΔN-HtrA3-ΔPDZ and ΔN-HtrA3S were monomeric, suggesting that the presence of the PDZ domain is necessary for trimer formation. The unique C-terminal sequence of HtrA3S had no apparent influence on activity or oligomerization status. In the case of ΔN-HtrA3, we were unable to demonstrate the formation of large multimers in the presence of a substrate, similar to those described for HtrA1 [9]. In addition, we examined the cleavage specificity of ΔN-HtrA3 via liquid chromatography–mass spectrometry (LC-MS) analysis of proteolysis products.

## Materials and Methods

### Materials

Fluorescent substrate Ala(Mca)IRRVSYSF–ANB–NH_2_ (where Mca is 7–methoxycoumarin–4–acetic acid and ANB–NH_2_ is the amide of 5–amino–2–nitro benzoic acid) whose sequence was based on the HtrA2 substrate described in [37], was synthesized and purified as described previously in [38]. Restriction enzymes and T4 ligase were purchased from Fermentas (Vilnius, Lithuania); primers used in site–directed mutagenesis were purchased from Genomed S.A. (Warszawa, Poland). β-casein from bovine milk, citrate synthase and malic dehydrogenase from porcine heart were from Sigma-Aldrich (Poznan, Poland). Other chemicals were purchased from Sigma-Aldrich or Fluka (Sigma-Aldrich, Poznan, Poland).

### Plasmid and strain construction

A fragment of an *HtrA3* cDNA encoding HtrA3 protein (amino acids 18–453) was amplified by PCR and cloned into *Nde*I and *Xho*I restriction sites of a pET24b (Novagen, San Diego, USA) plasmid. In a similar way a fragment of *HtrA3S* cDNA encoding HtrA3S isoform (amino acids 130–357) was cloned. The obtained constructs were used as the sources of *HtrA3* and *HtrA3S* genes in further PCR-based cloning into the expression plasmid pETcoco-2 (Novagen, San Diego, USA). The pPG1 (ΔN-HtrA3-His_6_) plasmid carried a DNA fragment, encoding HtrA3 amino acids 130–453 and an His_6_-tag at the C-terminus, incorporated into a pETcoco-2 vector via *Sph*I and *Not*I restriction sites. The pPG2N (His_6_-ΔN-HtrA3-ΔPDZ) plasmid carried a DNA fragment encoding amino acids 122–349 inserted into *Nhe*I and *Not*I sites of the pETcoco-2 vector, and this was a source of a ΔN-HtrA3-ΔPDZ protein with an His_6_-tag at the N-terminus. The pTW1 (ΔN-HtrA3S-His_6_) plasmid contained a DNA fragment encoding HtrA3S amino acids 130–357 cloned into *Sph*I and *Not*I sites of the pETcoco-2 vector, and was a source of the ΔN-HtrA3S protein with an His_6_-tag at the C-terminus. The S305A mutation of the *HtrA3* and *HtrA3S* genes was introduced by using site-directed mutagenesis with the Quick-Change Mutagenesis Kit (Stratagene). The primers used for cloning and mutagenesis are listed in Table 1. Plasmid sequences were verified by sequencing (Genomed S.A., Poland). The *E*. *coli* BL21(DE3) *clpP*::*Cm* ^*r*^ *s*train, deficient in ClpP protease activity, was obtained by P1*vir* phage transduction of the *clpP* mutation from *E*. *coli* C600 *clpP*::*cm* ^*r*^ [39] into *E*. *coli* BL21(DE3) (Novagen, San Diego, USA).

**Table 1:** Primers used to obtain *HtrA3* cDNA and construct the *HtrA3* gene variants.

Plasmid	Oligo-nucleotide	Orientation	Sequence (5’→3’)
*HtrA3* cDNA	NdeI	Forward	GCTTAACATATGCGGGAGCCCCCTGCGG
	BamHI	Reverse	GGAGGAATGGGATCCTCACATGACCACCTC
*HtrA3S* cDNA	NdeI	Forward	GTACATATGCACCAGCTGAGCAGCCCGCGC
	XhoI	Reverse	CCCTCGAGATGAACTGCCAGTGAGGGGGC
pETcoco-2 ΔN-HtrA3	SphI-F	Forward	TAAGCATGCTCCACCAGCTGAGCAGCCCG
	NotI-R	Reverse	TTGCGGCCGCTCAGTGGTGGTGGTGGTGG
pETcoco-2 ΔN-HtrA3-ΔPDZ	NheI-F	Forward	AAGCTAGCCAGAAGGGCGCCTGCCCGTTGG
	Stop-R	Reverse	GCGCTTCTTCCAGTCTTAGATCTGCTTGTC
pETcoco-2 ΔN-HtrA3S	SphI-F	Forward	TAAGCATGCTCCACCAGCTGAGCAGCCCG
	NotI-R	Reverse	TTGCGGCCGCTCAGTGGTGGTGGTGGTGG
S305A mutation	S305A-F	Forward	CCCCCGG**C**GTTCCCGTAGTTGATGATGG
	S305A-R	Reverse	CGGGAAC**G**CCGGGGGACCACTGGTG

The exchanged nucleotides are marked in **bold** format.

### Purification of proteins

*E*. *coli* BL21(DE3) *clpP*::*cm* ^*r*^ strain, transformed with appropriate plasmids was used to overproduce ΔN-HtrA3 (residues 130–453–His_6_), proteolytically inactive ΔN-HtrA3_S305A_, ΔN-HtrA3-ΔPDZ (residues His_6_–122–349), ΔN-HtrA3-ΔPDZ_S305A_, ΔN-HtrA3S (residues 130–357–His_6_) and ΔN-HtrA3S_S305A_ in a pET System (Novagen, San Diego, CA, USA). The proteins were purified by affinity chromatography on Ni-NTA columns according to the manufacturer’s instructions (Qiagen, Germany). The purity of the proteins was estimated to be more than 95% as judged by SDS-polyacrylamide gel electrophoresis. Prior to crystallization, the ΔN-HtrA3_S305A_ protein was further purified by gel-filtration on a HiLoad 16/60 Superdex 200pg column (GE Healthcare) in 10 mM HEPES buffer pH 7.5, 500 mM NaCl and 1 mM DTT. The protein was concentrated on Amicon Ultracel 10K centrifugal filters (Millipore) up to 16 mg/ml concentration. The concentration of the HtrA3 preparations was estimated by staining with Amido Black as described previously [40], or with the Bradford method [41] and, prior to protein crystallization, by using an ND-1000 Spectrophotometer System (Nanodrop Technologies).

### Protein crystallization

The initial crystallization condition was determined with a sparse crystallization matrix at the temperatures of 4°C and 16°C using the sitting-drop vapor-diffusion technique with an MCSG crystallization suite (Microlytic), and Pi-minimal and Pi-PEG screens [42] (Jena Bioscience). The best crystals were obtained after 4 months from C7 conditions of the MCSG4 screen (1 M sodium/potassium phosphate buffer pH 5.0) at 16°C. Crystals selected for data collection were soaked in the crystallization buffer supplemented with 25% glycerol and flash-cooled in liquid nitrogen.

### Data Collection, Structure Determination and Refinement

Single-wavelength X-ray diffraction data were collected at a temperature of 100 K at the 19-ID beamline of the Structural Biology Center [43] at the Advanced Photon Source at Argonne National Laboratory using the program SBCcollect. The intensities were integrated and scaled with the HKL3000 suite [44]. The structure was determined by molecular replacement using the HKL3000 suite [44] incorporating the MOLREP program [45] from the CCP4 suite [46]. The coordinates for the HtrA1 protein structure (PDB entry 3NZI) [9] modified by the CHAINSAW [47] program from the CCP4 suite [46] were used as the starting model for the PD domain search and the HtrA3 PDZ domain structure (PDB entry 2P3W) [12] was used for the PDZ domain search. Several rounds of manual adjustments to structure models were performed using COOT [48] and refinements with phenix.refine [49], implementing noncrystallographic symmetry (NCS) restraints, grouped B-factor refinement and automated TLS group assignment. The stereochemistry of the structure was validated with the PHENIX suite [49] incorporating MOLPROBITY [50] tools. Selected crystallographic statistics are provided in Tables 2 and 3. All protein structure images were generated using PyMOL version 1.5.0.3-Linux (Schrödinger LLC, www.pymol.com).

**Table 2:** Crystal data collection statistics.

X-ray wavelength (Å)	0.9792
Space group	P4_3_2_1_2
Unit cell dimensions	a = b = 119.0Å, c = 167.9 Å, α = β = γ = 90°
Resolution* (Å)	40.8 – 3.27 (3.33–3.27)
No. of unique reflections	18953 (927)
Completeness	98.4% (99.8%)
R-merge	0.085 (0.86)
R-pim	0.028 (0.274)
CC1/2 (Å^2^)	- (0.76)
I/σ	8.7 (2.72)
Redundancy	9.6 (9.7)
Wilson plot B-factor (Å^2^)	109.4
Molecules per asymmetric unit	3
No. of protein residues	993

* Numbers in parentheses are shown for the highest resolution shell.

**Table 3:** Structure refinement statistics.

Resolution range (Å)	40.8–3.27 (3.44–3.27)
Reflections	18907 (2637)
σ cutoff	None
R-value (all) (%)	20.1
R-value (R-work) (%)	19.9 (26.6)
Free R-value (%)	23.3 (31.7)
**Rms deviations from ideal geometry**	
bond length (Å)	0.003
angle (degrees)	0.83
chiral (Å)	0.03
**No. of atoms**	
Protein	5367
phosphate ion	10
Water	3
**Mean B-factor (Å** ^**2**^ **)**	
all atoms	112.7
protein atoms	112.6
protein main chain	110.0
protein side chain	115.5
phosphate ion	146.1
Water	67.1
**Molprobity Ramachandran plot statistics**	
Residues in favored regions (%)	95.9
Residues in allowed regions (%)	99.9
Residues in disallowed region (%)	0.1

### Coordinates

Atomic coordinates and structure factors were deposited into the Protein Data Bank as 4RI0.

### Analysis of the protease activity

The HtrA3 protease activity using β-casein as a substrate was analyzed within the temperature range of 25–45°C (every 5°C) as described in [51]. ΔN-HtrA3, ΔN-HtrA3-ΔPDZ or ΔN-HtrA3S (0.14 μM) were incubated with β-casein (28 μM) in 25 mM HEPES pH 7.5 buffer, 100 mM NaCl, in a final volume of 180 μl (the final enzyme: β-casein molar ratio was 1: 200). Samples were withdrawn every 5 minutes, for 45 min. The reaction was terminated by the addition of Laemmli lysis buffer and immediate freezing at –20°C. The samples were then resolved by 12.5% SDS-PAGE and gels were stained with Coomassie Brilliant Blue. The electrophoregrams were analyzed densitometrically using the 1DScan EX version 3.0.2 Eval (Scanalytics Inc., USA, www.scanalytics.com). ΔN-HtrA3, ΔN-HtrA3-ΔPDZ and ΔN-HtrA3S proteolytic activity with the fluorescent substrate Ala(Mca)IRRVSYSF–ANB–NH_2_ was assayed as described in [38]. The reaction was carried out in the presence of 10 nM enzyme, 5 μM substrate peptide, in 50 mM Tris-HCl pH 8.0, 10% glycerol buffer at temperatures 25–45°C (every 5°C) for 15 min and fluorescence was measured as a function of time, using a Perkin Elmer LS55 luminescence spectrometer connected to a Julabo F12 water bath, with excitation at 315 nm and emission at 400 nm. The linear region of the fluorescence versus time plot was used to calculate the initial reaction rate. The data were fitted by linear regression using OriginPro 9.1 software (OriginLab Corp., USA, www.originlab.com). The activity was calculated using a standard curve prepared with Fmoc–β–(7–methoxy–coumarin–4–yl)–Ala–OH at temperatures 25–45°C (every 5°C). In kinetic measurements, the fluorescent substrate was used at concentrations ranging from 0.02 to 6 μM, the enzyme at 50 nM concentration, and fluorescence was measured at 30°C as a function of time. The initial reaction rate was calculated as described above. Enzyme concentrations refer to monomers. At least three independent measurements were performed and the standard deviation did not exceed 10%. Steady-state kinetic parameters were obtained by fitting the data to the Michaelis-Menten equation using GraphPad Prism 5 software (GraphPad Software, Inc., USA, www.graphpad.com).

### Digestion of model substrates and identification of the products

The degradation of substrate proteins was performed by incubating 0.14 μM ΔN-HtrA3 in 25 mM HEPES buffer (pH 7.5) with 28 μM β-casein at 37°C or 0.56 μM ΔN- HtrA3 in 100 mM Tris-HCl buffer (pH 8.5), 100 mM NaCl with 28 μM denatured malic dehydrogenase at 30°C. Malic dehydrogenase denaturation was conducted by incubation at 43°C for 10 min prior to digestion. ΔN-HtrA3 incubated without substrate served as a control. Samples were taken at 5, 10, 15, 30 and 45 min in triplicates. Two samples were frozen in liquid nitrogen and one was mixed with an equal volume of SDS loading buffer and analyzed by SDS-PAGE. The samples in which there was no visible degradation of ΔN-HtrA3, the control samples without the enzyme and control ΔN-HtrA3 incubated without substrate were subjected to further analysis. The cleavage products were identified via mass spectrometry technique using a LC-MS (ESI) MS-Bruker Daltonics HCT Ultra coupled with LC-Agilent Technologies 1200 series. The linear gradient of phase A (0.1% HCOOH in H_2_O) to B (0.1% HCOOH in 80% ACN) was performed within 60 minutes. An Agilent Eclipse ADB-C8 column, 5 μm, 4.6×150 mm was used. The flow rate was equal to 0.8 ml/min. UV detection was at 226 nm and 254 nm. A digestion pattern and sequence search was performed using the Mascot data base. The peptide sequences were identified based on the m/z signals. The ΔN-HtrA3 self-cleavage products were used as a control and the identified peptides were not included in the final analysis. The ΔN-HtrA3 concentrations refer to monomers.

### Size exclusion chromatography

SEC was performed at room temperature with an Agilent High Performance Liquid Chromatography (HPLC) system using a Superose 12 HR 10/30 column (GE Healthcare Life Sciences). A sample of 50 μl was loaded into the column which had been preequilibrated with buffer containing 50 mM Na_2_HPO_4_/NaH_2_PO_4_ pH 8.0 and 250 mM NaCl. The concentration of HtrA3 proteins in the analyzed samples was 3 mg/ml unless stated otherwise. When the HtrA3-substrate complexes were analyzed, 25 μl of 32 μM (trimer) ΔN-HtrA3_S305A_ protein was incubated with 25 μl of 50 μM β-casein or 25 μl of 150 μM citrate synthase, or with 25 μl of 150 μM malic dehydrogenase (for 15 min at 30°C). Citrate synthase and malic dehydrogenase were denatured at 43°C for 10 min prior to incubation with HtrA3 protein. To analyze the HtrA3–peptide complexes, β-casein was hydrolyzed with ΔN-HtrA3 and the hydrolysate was used in SEC as described in [52]. Proteins were eluted at a flow rate of 0.3 ml/min. The standards used for calibration included thyroglobulin, apoferritin, β–amylase, alcohol dehydrogenase, bovine serum albumin, ovalbumin, carbonic anhydrase and vitamin B12 (Sigma-Aldrich).

### Protein crosslinking

Crosslinking of the proteins was performed using Bis(sulfosuccinimidyl) suberate (BS^3^), as described previously [53].

## Results

### Structure of the HtrA3 protease-PDZ domains

We expressed in a bacterial system, purified and crystallized the ΔN-HtrA3 (residues 130–453) with a mutated catalytic residue (S305A). This mutation eliminated the serine protease activity, which was responsible for the partial degradation of the ΔN-HtrA3 protein at the high concentrations required for crystallization. The best crystal diffracted to a resolution of 3.27 Å and belonged to the tetragonal space group P4_3_2_1_2 (Table 2). The X-ray diffraction was very clean with low background and well separated rounded diffraction spots, slightly elongated at higher resolution. The structure was solved by the molecular replacement method using the HtrA1 PD [9] and the HtrA3 PDZ [12] domains as search models. To compensate for the limited amount of experimental data due to low resolution, we applied several refinement restraints in the phenix.refine program [49]. These were group B-factor and TLS group refinement, secondary structure and noncrystallographic symmetry (NCS) (for the PD domains) restraints, as well as optimization of the weights between X-ray target and stereochemistry. The final round of refinement converged to the R factor of 20.1% and the R_free_ value of 23.3% (Table 3). The unexpectedly low R-factor values most likely result from the very good quality of diffraction and the high symmetry space group of the crystal. On the other hand, the final model exhibits very high Atomic Displacement Parameters (ADPs or B-factors) with a mean value of 112.6 Å^2^ for all protein atoms. This is a result of the high Wilson B factor of the collected data (109 Å^2^), which is often observed for low-resolution data sets.

**Fig 1:**
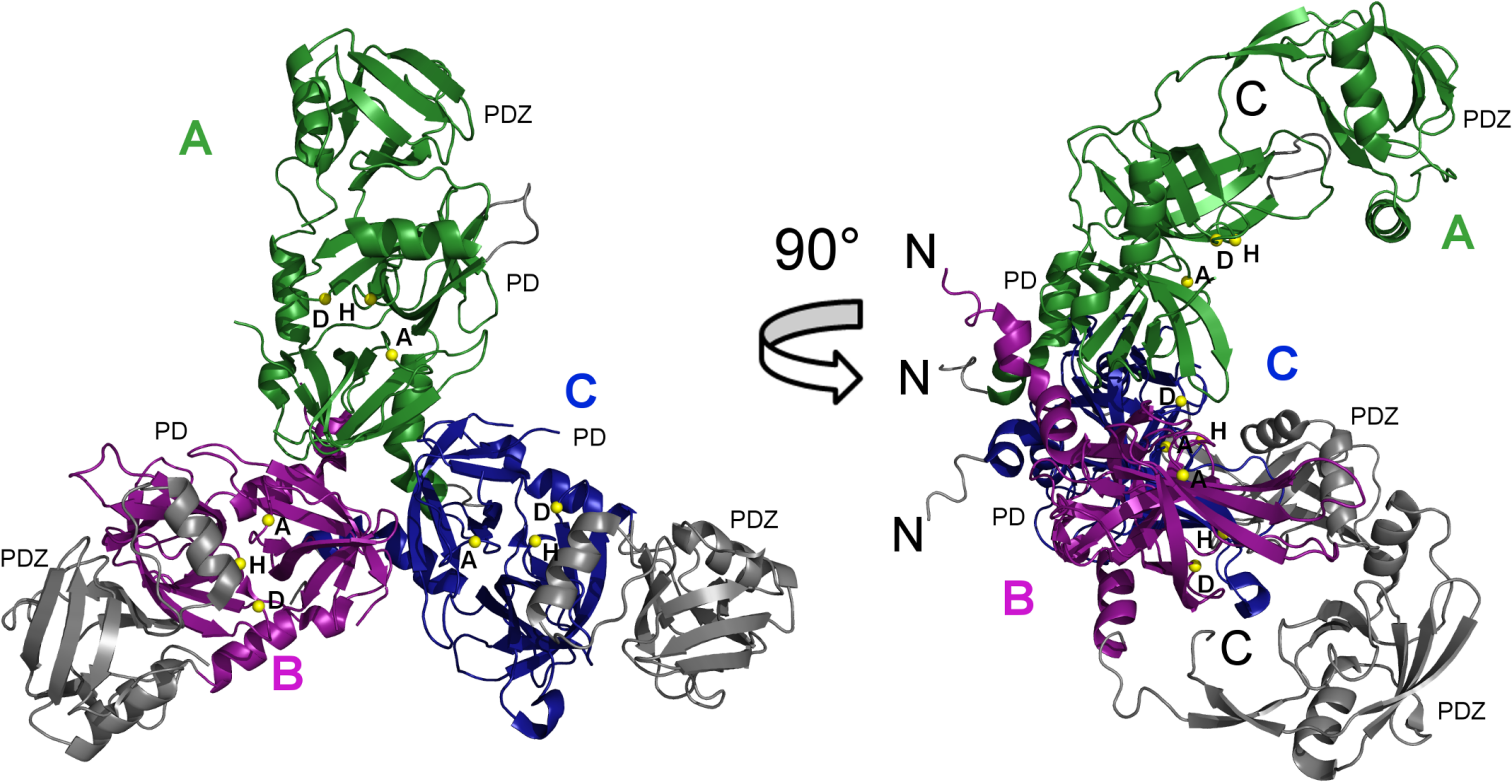
The ΔN-HtrA3 trimer structure. Reconstruction of the ΔN-HtrA3_S305A_ trimer based on the X-ray crystal structure determined in this study. The monomers (A, B and C) are shown in separate colors. The serine protease (PD) and PDZ domain in each monomer are labeled. Missing PDZ domains of chains B and C were modeled based on the PDZ domain of chain A. Also, the missing chain A loop (residues163-168) was modeled based on the loop in chain B. The modeled regions are colored gray. The location of the catalytic residues H191, D227 and S305A in each monomer are marked with yellow balls and the residues labeled H, D, A. The N- and C-termini are labeled N and C, respectively, in black. C-terminal cloning-artifact residues (His-tag) were omitted.

**Fig 2:**
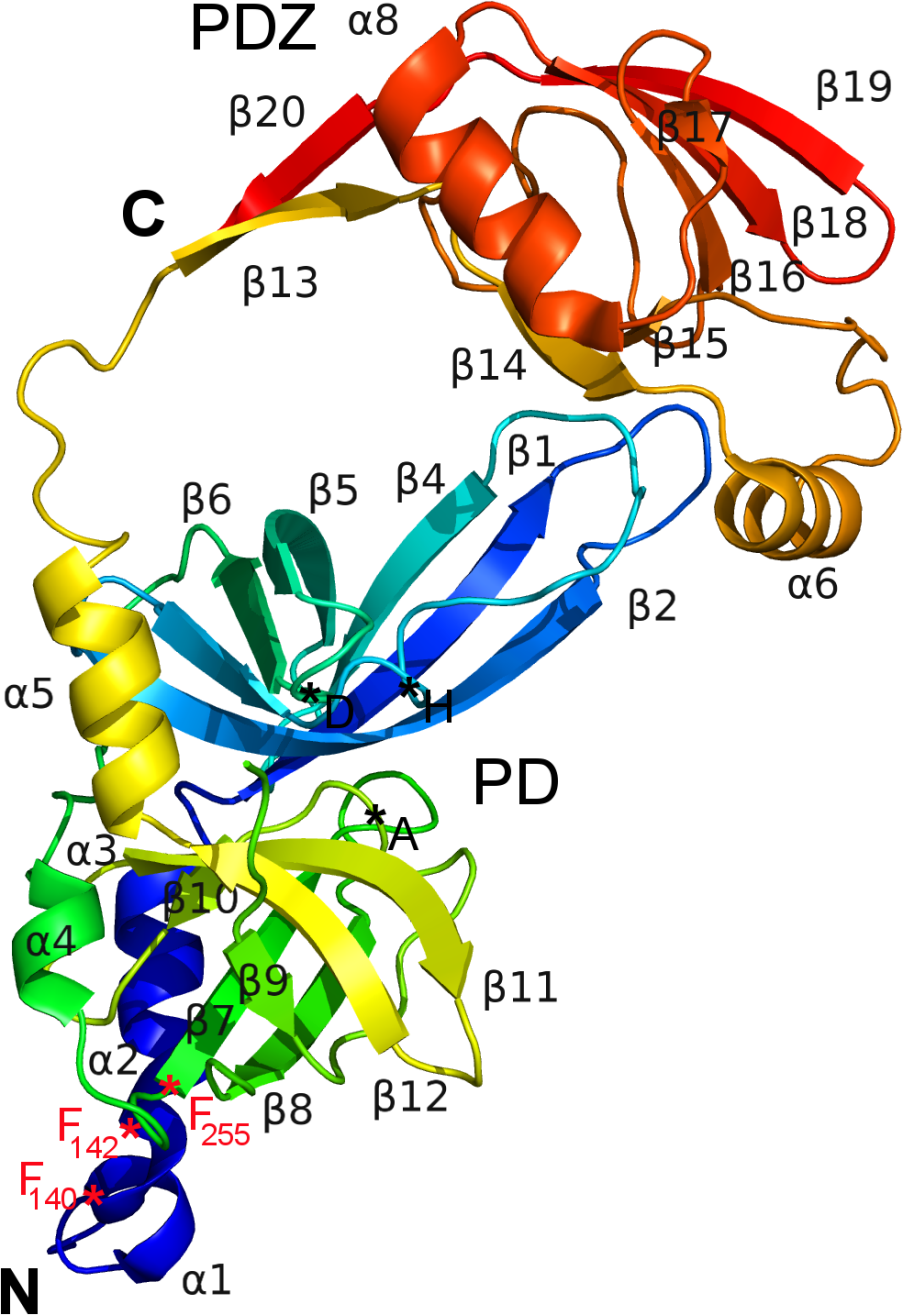
The ΔN-HtrA3 monomer (chain A) structure. Rainbow coloring from blue to red indicates the N- to C-terminal position of the residues in the model. The location of the catalytic (H, D, A) and the interlocking F140, F142 and F255 residues are indicated by the asterisks.

**Fig 3:**
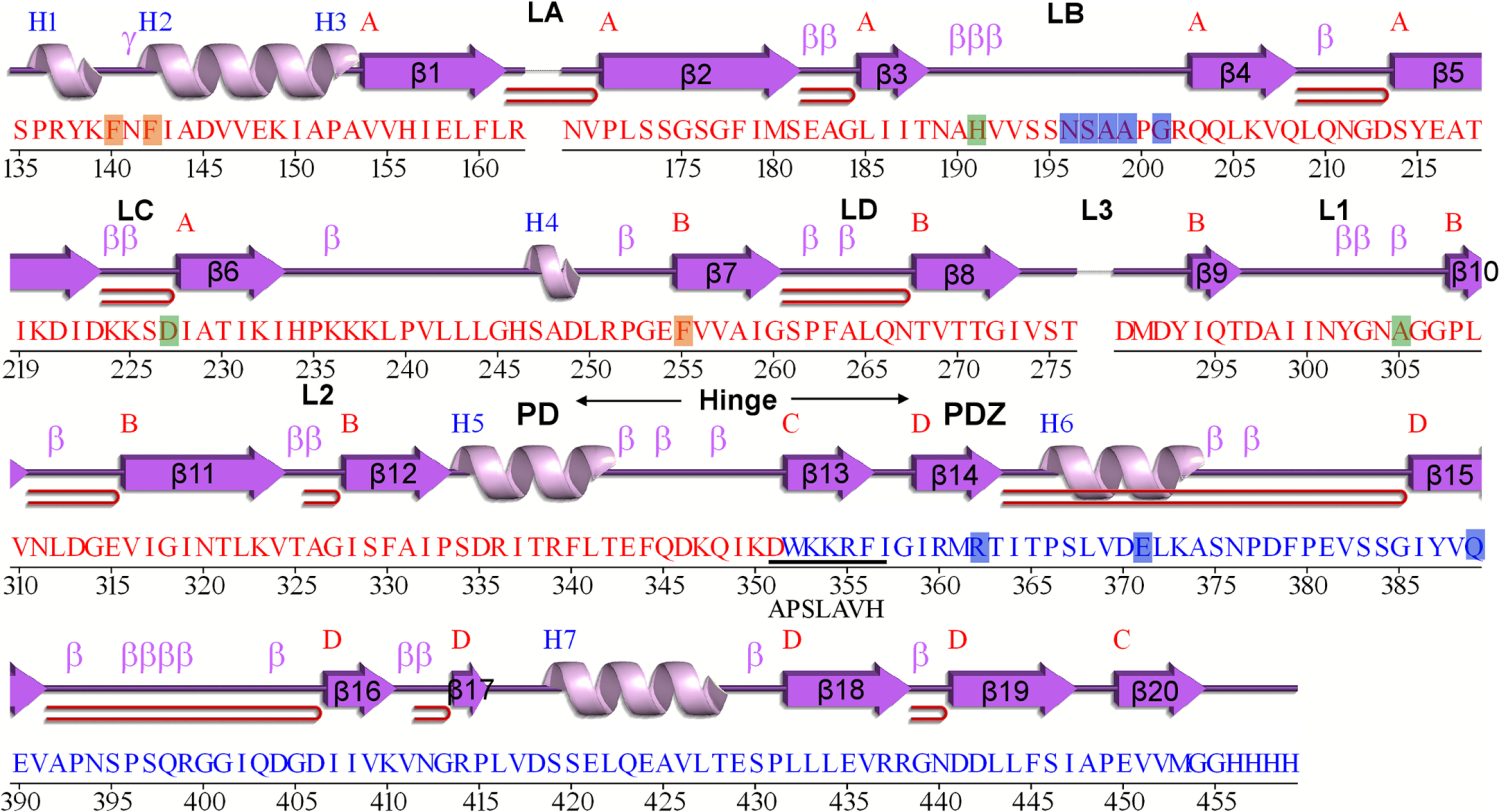
Secondary structure of the ΔN-HtrA3 protein chain A as defined by the PDBSum server (http://www.ebi.ac.uk/pdbsum/). α-helices are labeled H1, H2, …, H8 and β-strands by their sheets as A, B, C, and D. Structural motifs β-turns, γ-turns, and β-hairpins are marked as β, γ, and ⊃, respectively. The catalytic residues H191, D227 and S305A are marked in green, phenylalanine residues involved in trimerization are orange, and residues involved in LB loop – PDZ interactions are blue. C-terminal amino acids (residues 351–357) of the ΔN-HtrA3S isoform are shown below the ΔN-HtrA3 sequence (underlined). The amino acid sequence 1–350 of HtrA3S is identical to that of HtrA3L.

**Fig 4:**
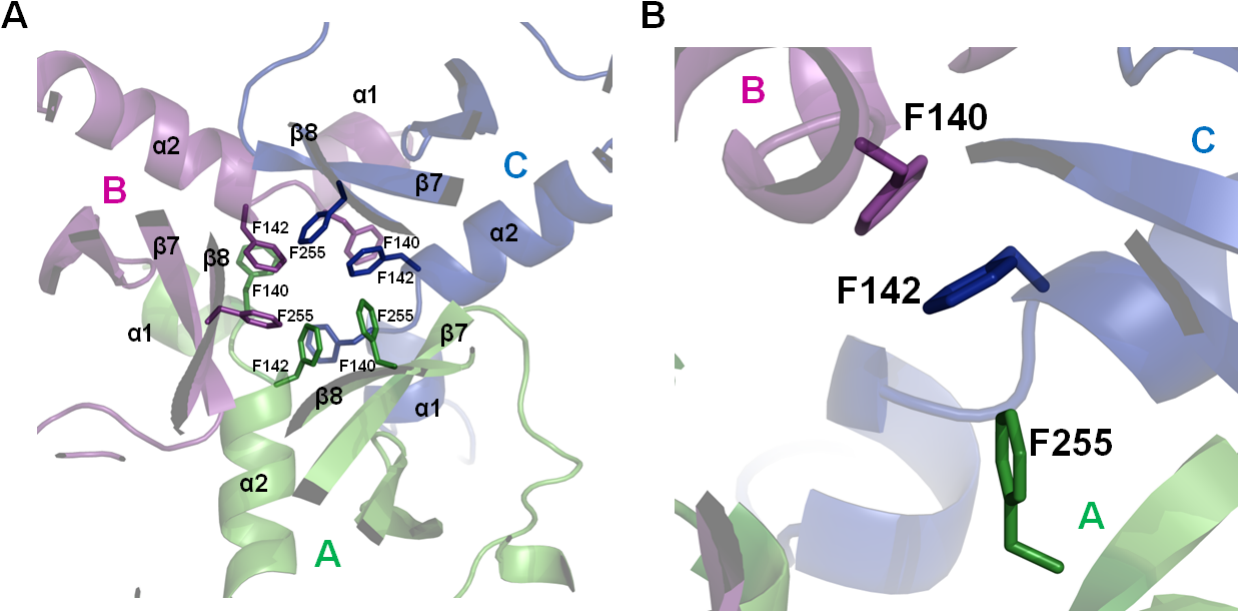
The key residues involved in HtrA3 trimer formation. (A) The trimer is stabilized by three phenylalanines in the PD of each monomer. (B) A “lock201D structure formed by F140, F142 and F255. The HtrA1 protein PD has an analogous trimer arrangement with F140 replaced by tyrosine. The chains A, B and C of the ΔN-HtrA3 trimer are shown in green, magenta and blue, respectively.

The structure of the ΔN-HtrA3_S305A_ protein (Figs 1, 2 and 3) closely resembles the structures of two other human HtrA proteins: HtrA1 and HtrA2 [9] [11] [12]. The asymmetric unit of the ΔN-HtrA3 crystals contains three molecules of the monomeric protein which represents the trimeric HtrA3 biological assembly. The trimer is stabilized by the interaction of three phenylalanines (F140, F142, and F255) from each monomer, forming a “lock” between monomers similar to other HtrA proteins [9] (Fig 4). The only difference in the “lock” structure is the presence of HtrA3 F140 in place of a tyrosine in both HtrA1 and HtrA2. Chain A is the only molecule visible in full length in the ΔN-HtrA3 structure. It encompasses residues 135–459 with the exclusion of residues 163–168 and 277–289 of the loops LA and L3, respectively. The loops are named according to the chymotrypsin nomenclature (Fig 3) [54]. Two other chains encompass only PDs, residues 130–344 for chain B and 134–344 for chain C. Owing to the lack of electron density, the PDZ domains in the B and C chains are missing in the final structure, probably as a result of multiple domain positions inside the crystal. The PD-PDZ domain conformation observed in chain A could be one of several conformations for the chain B and must be excluded in the case of the chain C molecule due to the crystal packing. The chain C PDZ domain must have a different position than the one defined in chain A to avoid a collision with neighboring molecules inside the crystal and has to be moved to a vacant space (the ΔN-HtrA3_S305A_ crystal solvent content is 55%). A similar situation was observed for the HtrA1_S328A_ protein structure where only the PDs were modeled and all PDZ domains were undefined (PDB entry 3NUM) [9].

**Fig 5:**
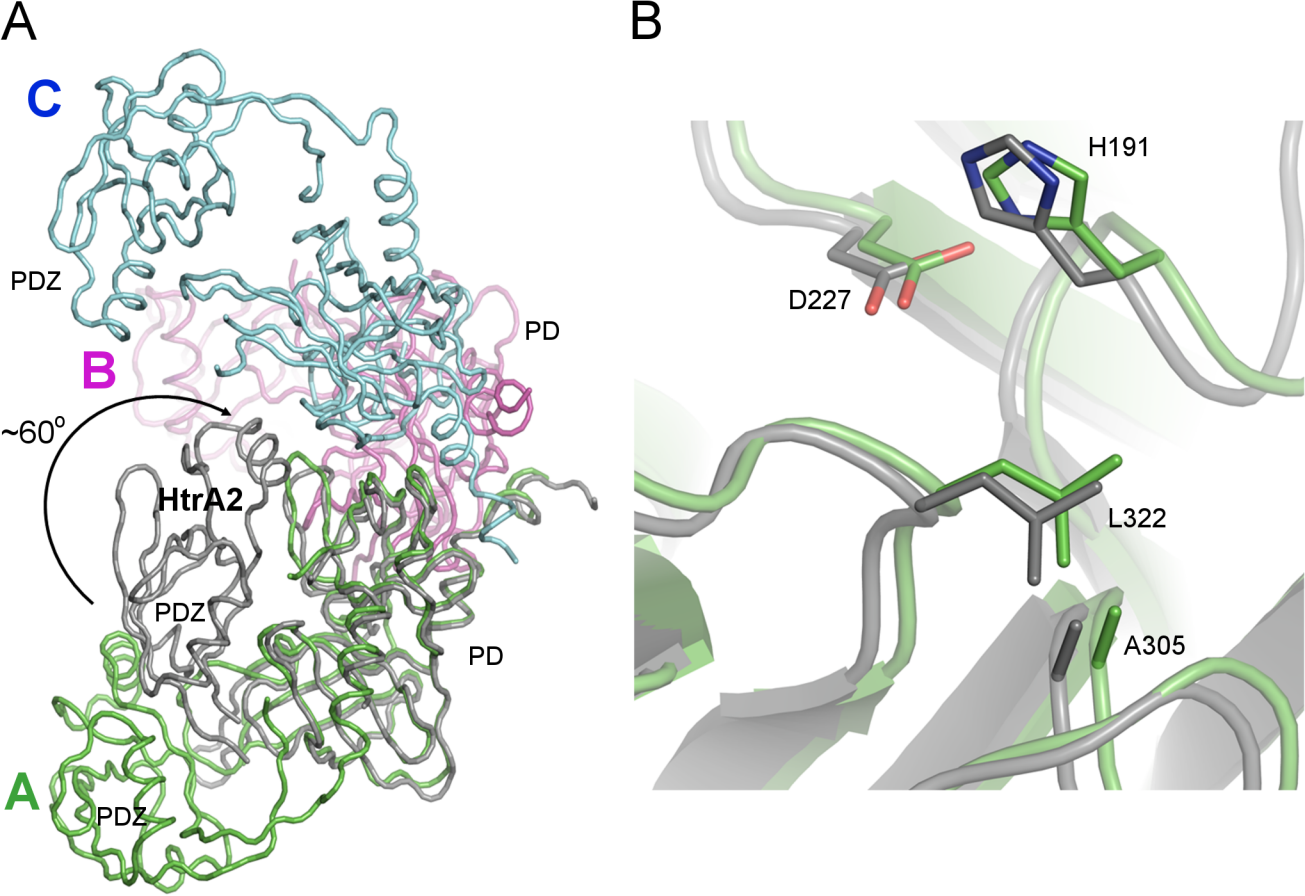
Comparison of the HtrA3 structure with the structures of HtrA1 and HtrA2. (A) Superposition of ΔN-HtrA3 (green) and HtrA2 (gray) (PDB entry 1LCY) monomers. The structures were aligned via the Cα atoms of the PDs. The HtrA2 structure has a closed conformation whereby the PDZ domain has rotated ~60^0^ around the interdomain linker (direction indicated by the arrow). The subunits of the ΔN-HtrA3 trimer are shown in green, magenta and blue. (B) Superposition of the ΔN-HtrA3_S305A_ (green) and HtrA1_S328A_ (PDB entry 3TJO, gray) catalytic sites. The residues of the catalytic triad are shown as sticks. The residue numbers correspond to the HtrA3 protein. The ΔN-HtrA3 catalytic triad is not properly positioned, with S305A too distant from H191 (9.5 Å) for proton transfer. The residue L322 occludes the S1 specificity pocket.

The reconstructed ΔN-HtrA3 trimer structure based on the chain A molecule has a conical shape with peripheral PDZ domains pointing away from the center of the trimer (Fig 1). The ΔN-HtrA3 trimer structure appears to be in an intermediate conformation, lying between the flat saucer-like HtrA1 SAXS structure (“open form”) [10] and the compact pyramidal HtrA2 X-ray structure (“closed form”) [11]. The change of trimer shape from the ΔN-HtrA3 conformation to one similar to HtrA2 will require a rotation of the PDZ domain around the interdomain linker (residues 350–352) by about 60^0^ (Fig 5A).

**Fig 6:**
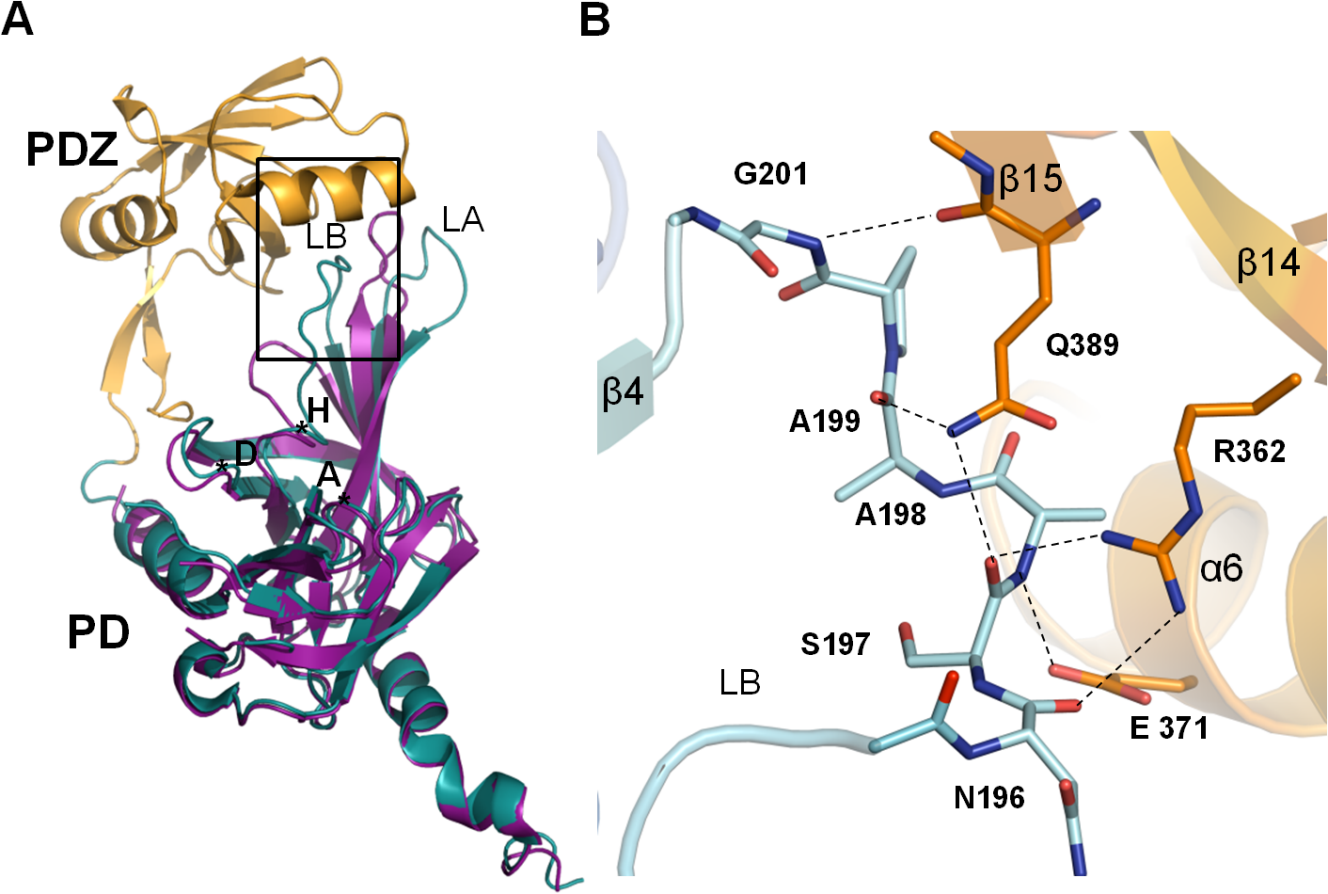
Interaction of the ΔN-HtrA3 LB loop with the PDZ domain. (A) Superposition of ΔN-HtrA3 (PD in blue, PDZ domain in orange) (this work) and HtrA1 (magenta, PDB entry 3TJO) monomer structures. The LA and LB loops are indicated. (B) A blowup (with a slight rotation for picture clarity) of the superposition highlighting the interaction between the ΔN-HtrA3 LB loop (residues 196–201) with the PDZ domain. The essential residues are shown as sticks. Hydrogen bonds are shown as black dotted lines.

The ΔN-HtrA3 monomer structure (Fig 2) contains two distinct domains, the PD and PDZ. The PD adopts a chymotrypsin fold and is formed by two 6-strand β-barrels flanked by four α-helices (Figs 1, 2 and 3). The closest structural homolog for the domain is HtrA1_S328A_ PD (PDB entry 3TJO) [10]. The two structures have a DALI [55] Z-score value (strength of structural similarity in standard deviations above expected) of 33 and an RMSD (root mean square deviation of superimposed Cα atoms in Å) equal to 1.0 for 197 residues. Both structures have nearly the same fold, however there are some differences in external loops, particularly residues 161–170 and 194–204, (loops LA and LB, respectively; Fig 3). The LB loop is six residues longer than the analogous HtrA1 and HtrA2 loops. The main-chain nitrogen and oxygen atoms of the loop form multiple hydrogen bonds with PDZ residues R362, E371, and Q389, generally in the distance range of 3.0–3.2 Å (Fig 6).

The ΔN-HtrA3 active site is virtually the same as in HtrA1_S328A_ (Fig 5B). The catalytic triad, H191-D227-S305A, is placed identically to the HtrA1_S328A_ structure. Also, all other residues involved in peptide binding are the same and are positioned in a similar way [9]. The access to the active site is open and not blocked by the PDZ domain as in HtrA2 [11].

The X-ray structure of the HtrA3 PDZ domain at a resolution of 1.7 Å has been reported and described previously [12]. Our structure of the PDZ domain is practically the same as the published one. Surprisingly, we were able to model the domain as a continuous and complete amino-acid chain without the breaks present in the high-resolution structure at residues 372–381.

### ΔN-HtrA3 oligomerization status in the presence of a substrate

**Fig 7:**
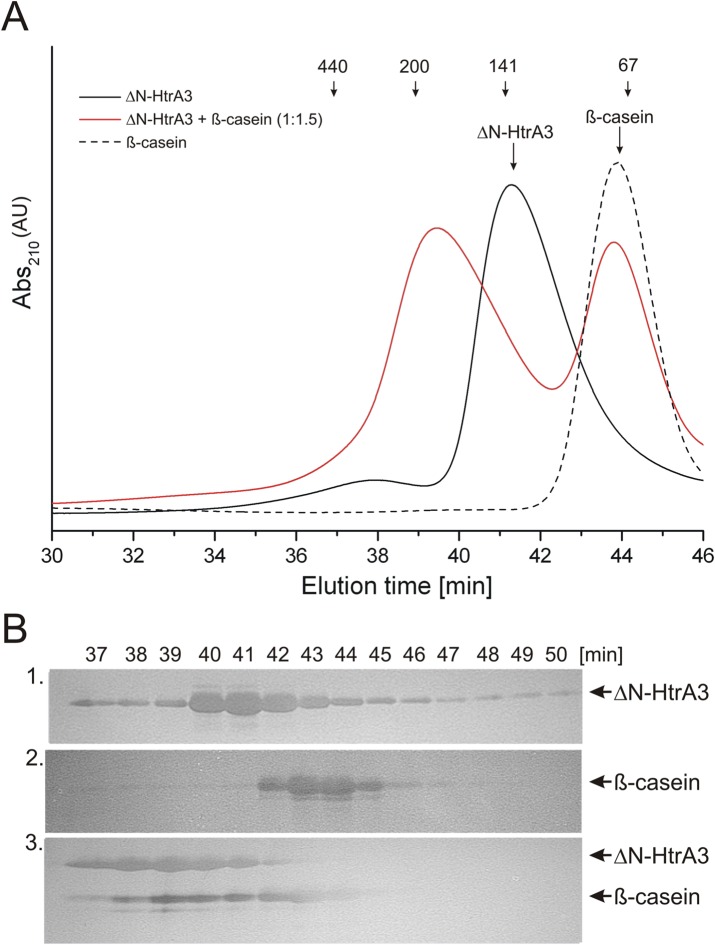
ΔN-HtrA3 oligomerization state in the presence of substrate. (A). Proteolytically inactive ΔN-HtrA3_S305A_ (black solid line) and β-casein (black dashed line) were incubated at the ΔN-HtrA3 trimer to β-casein (monomer) ratio of 1:1.5 (red) and fractionated by size exclusion chromatography. (B) Peak fractions of ΔN-HtrA3, β-casein, and ΔN-HtrA3–β-casein complex were resolved using SDS-PAGE and silver stained. The faintness of the β-casein bands in fractions 43 and 44 in panel B3 is most probably due to a poorer staining.

In HtrA proteases, substrate binding can coincide with conversion from lower to higher oligomers, for example 12- or 24-mers [1] [56] [57]. Size exclusion chromatography (SEC) of HtrA1 (composed of PD and PDZ domains) in the presence of a substrate indicated that HtrA1 assembles as a large multimer of ~600 kDa [9]. To investigate the oligomerization status of ΔN-HtrA3 in solution and the influence of a substrate on oligomerization, we incubated proteolytically inactive ΔN-HtrA3_S305A_ with the unfolded substrate β-casein and analyzed the samples via SEC. The results (Fig 7 and Table 4) showed that ΔN-HtrA3 eluted as ~120 kDa protein, suggesting that it is trimeric in solution (the theoretical molecular mass of ΔN-HtrA3 trimer is 109.3 kDa). In the presence of β-casein, the major ΔN-HtrA3 fraction eluted as ~170 kDa protein and the peak fractions contained β-casein (Fig 7B), suggesting that the ΔN-HtrA3 protein bound the substrate. The theoretical molecular mass of ΔN-HtrA3 hexamer is 218.6 kDa and that of the hexamer with one molecule of β-casein is 231 kDa. Hence, the observed complex appears to be smaller than the hexamer. Thus, under comparable experimental conditions ΔN-HtrA3 did not seem to form large multimers of size similar to those of HtrA1. Use of different substrates (unfolded malic dehydrogenase and citrate synthase, or the peptides obtained after digestion of β-casein with HtrA3) did not allow us to visualize any higher oligomeric forms. Also, employment of other techniques, protein crosslinking with a substrate peptide (RRVSYSF) or ultracentrifugation, was unsuccessful in visualization of welldefined higher order oligomers of ΔN-HtrA3 (results not shown).

**Table 4:** Size exclusion chromatography of HtrA3 protein variants.

Protein	Theoretical molecular mass [kDa]	Calculated molecular mass [kDa]
**ΔN-HtrA3**	109.3 (trimer)	123.2 ±6.2
**β-casein**	72 (trimer)	70±0.7
**ΔN-HtrA3 + β-casein (1:1.5)**	-	170.5±9.6
**ΔN-HtrA3-ΔPDZ**	25.8 (monomer)	34.5±0.1
**ΔN-HtrA3S**	25.6 (monomer)	33.4±3.0

Calculated molecular mass is an average of at least three independent experiments. ΔN-HtrA3 to β-casein molar ratio was calculated for ΔN-HtrA3 trimers. All HtrA3 variants had the S305A substitution and were proteolytically inactive.

### Role of the PDZ domain in proteolysis and oligomerization

**Fig 8:**
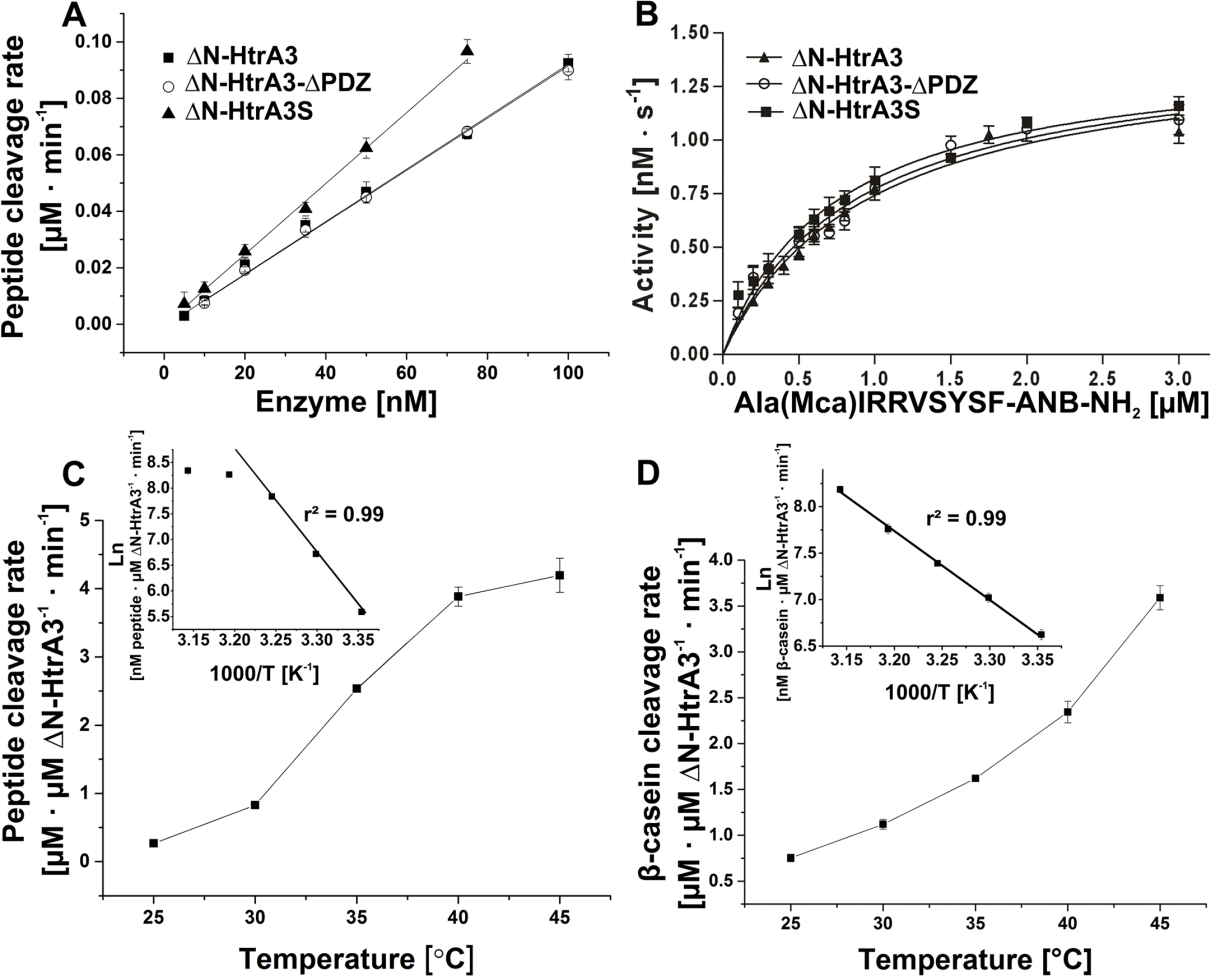
Effect of the PDZ domain upon ΔN-HtrA3 proteolytic activity. (A) Rates of cleavage of the peptide Ala(Mca)IRRVSYSF-ANB-NH_2_ (5 μM) by ΔN-HtrA3, ΔN-HtrA3-ΔPDZ, and ΔN-HtrA3S at increasing enzyme concentrations at 30°C. (B) Concentration dependence of the rate of peptide cleavage by ΔN-HtrA3, ΔN-HtrA3-ΔPDZ and ΔN-HtrA3S (50 nM) at 30°C. The data were fitted to the Michaelis-Menten equation using GraphPad Prism 5. (C) The temperature dependence of ΔN-HtrA3 activity, assayed with saturating amount of peptide (5 μM). (D) Temperature dependence of ΔN-HtrA3 activity, assayed with β-casein. The insets in C and D show Arrhenius plots of the same data. The data were fitted by linear regression using OriginPro 9.1 software. ΔN-HtrA3 concentrations were calculated for monomers. Error bars are averages +/ SD (*n* = 3).

In earlier studies, HtrA3L and HtrA3S showed similar levels of protease activity with β-casein as a substrate, suggesting that PDZ domain was not required for HtrA3 activity. Also, both proteins had an autoproteolytic activity at 17°C but not at 4°C [35] [58]. However, the classical kinetic analyses had not been performed, thus it was not known whether the PDZ domain would influence the protease substrate affinity. A possible role of the PDZ domain in the temperature dependence of the protease activity towards external substrates had also not been fully investigated. Thus, we decided to study the function of the PDZ domain in proteolysis in more detail. We assayed the activity of ΔN-HtrA3, ΔN-HtrA3 with removed PDZ domain (i.e., ΔN-HtrA3-ΔPDZ), and ΔN-HtrA3S, the N-terminally deleted natural HtrA3 isoform lacking PDZ, using a fluorogenic peptide Ala(Mca)IRRVSYSF-ANB-NH_2_ [38] and unfolded β-casein. The peptide sequence was based on that of the HtrA2-Opt peptide, a specific substrate of HtrA2 protease [37]. Using RP-HPLC and mass spectrometry methods, we found that ΔN-HtrA3 cleaved the Ala(Mca)IRRVSYSF-ANB-NH_2_ peptide between V and S, similarly as HtrA2 (results not shown). The HtrA3 variants cleaved the peptide very efficiently, at the rate proportional to the amount of the enzyme. Replacing the catalytic serine with alanine in all HtrA3 protein variants completely eliminated the peptide cleavage (data not shown). Plot of the initial velocities as a function of ΔN-HtrA3 concentration showed a linear relationship with a slope of 0.0009 μM product min^-1^ nM^-1^ enzyme (Fig 8A). The slope for ΔN-HtrA3-ΔPDZ was identical (0.0009 μM product min^-1^ nM^-1^ enzyme) and the slope for ΔN-HtrA3S was a little higher (0.0012 μM product min^-1^ nM^-1^ enzyme) compared to that of ΔN-HtrA3, indicating that the PDZ domain was not required for the enzymatic activity of ΔN-HtrA3. Peptide cleavage by ΔN-HtrA3 followed classical Michaelis-Menten kinetics; the kinetic parameters of ΔN-HtrA3-ΔPDZ were almost identical and those of ΔN-HtrA3S were similar, with a slightly decreased *K*_m_ value and an increased *k*_cat_/*K*_m_ value (Fig 8B and Table 5). These data indicate that the lack of the PDZ domain did not impair substrate affinity or turnover rate of the enzyme, and also suggest that the ΔN-HtrA3S isoform had a slightly increased affinity for the substrate. The latter could be due to the presence of the unique seven C-terminal amino acids in ΔN-HtrA3S (shown in Fig 3). This however needs further clarification. The kinetic values of μM product min^-1^ nM^-1^ enzyme were similar to those observed previously for HtrA2 [38]; however, in contrast to HtrA2, the ΔN-HtrA3 kinetics did not indicate the presence of allosteric regulation. Compared to HtrA1, whose activity at 37°C with a very similar peptide was ~20 nM product min^-1^ nM^-1^ enzyme [10], the ΔN-HtrA3 activity (~3 nM product min^-1^ nM^-1^ enzyme) was approximately 7-fold lower (Fig 8C and results not shown).

**Table 5:** Steady state kinetic parameters for HtrA3 protein variants with the peptide Ala(Mca)IRRVSYSF-ANB-NH_2_ as the substrate.

HtrA3 Proteins	K_m_ [μM]	k_cat_ [s^-1^]	k_cat_/ K_m_ [M^-1^ • s^-1^]
**ΔN-HtrA3**	0.958	0.029	30271
**ΔN-HtrA3-ΔPDZ**	0.878	0.029	33030
**ΔN-HtrA3S**	0.740	0.030	40502

**Fig 9:**
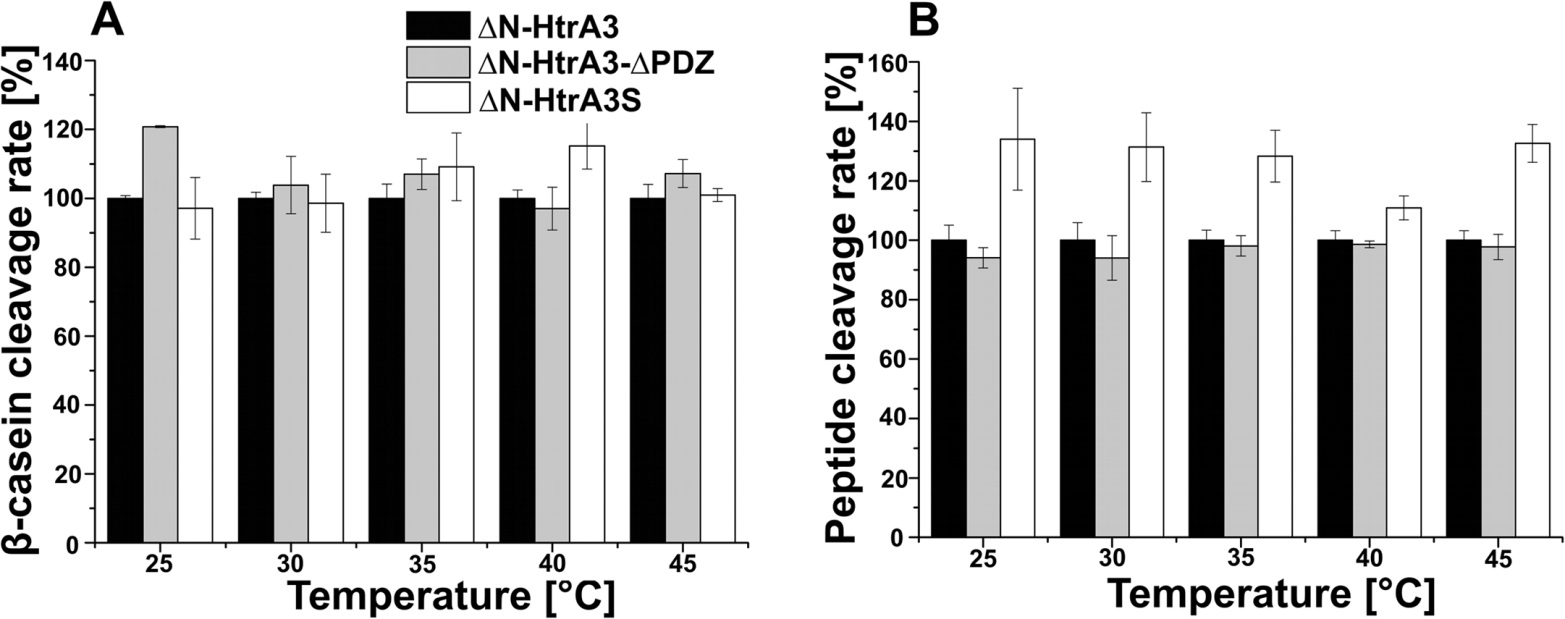
Influence of the PDZ domain on ΔN-HtrA3 activity at different temperatures. ΔN-HtrA3, ΔN-HtrA3-ΔPDZ, and ΔN-HtrA3S were assayed with (A) β-casein and (B) peptide Ala(Mca)IRRVSYSF-ANB-NH_2_ as substrates. The activity of ΔN-HtrA3 at a given temperature has been normalized to 100%. Error bars are averages +/ SD (*n* = 3).

ΔN-HtrA3 activity was highly dependent on temperature: it was practically negligible at 20°C (not shown) and increased approximately 5-fold between 25 and 45°C (Fig 8C and 8D). The temperature dependence of the activity assayed with the peptide substrate and presented as an Arrhenius plot suggested that the ΔN-HtrA3 protein might become unstable at 40–45°C (Fig 8C). At all temperatures tested (25–45°C), the activity of ΔN-HtrA3-ΔPDZ was similar to that of ΔN-HtrA3 while the activity of ΔN-HtrA3S was either similar (with β-casein) or a little higher (with peptide) (Fig 9A and 9B), showing that the PDZ domain has no apparent effect on the enzymatic activity of ΔN-HtrA3 at a wide range of temperatures. Furthermore, since ΔN-HtrA3S differs from ΔN-HtrA3-ΔPDZ only by the presence of the additional 7 C-terminal residues, these residues appear to have little effect on protease activity.

**Fig 10:**
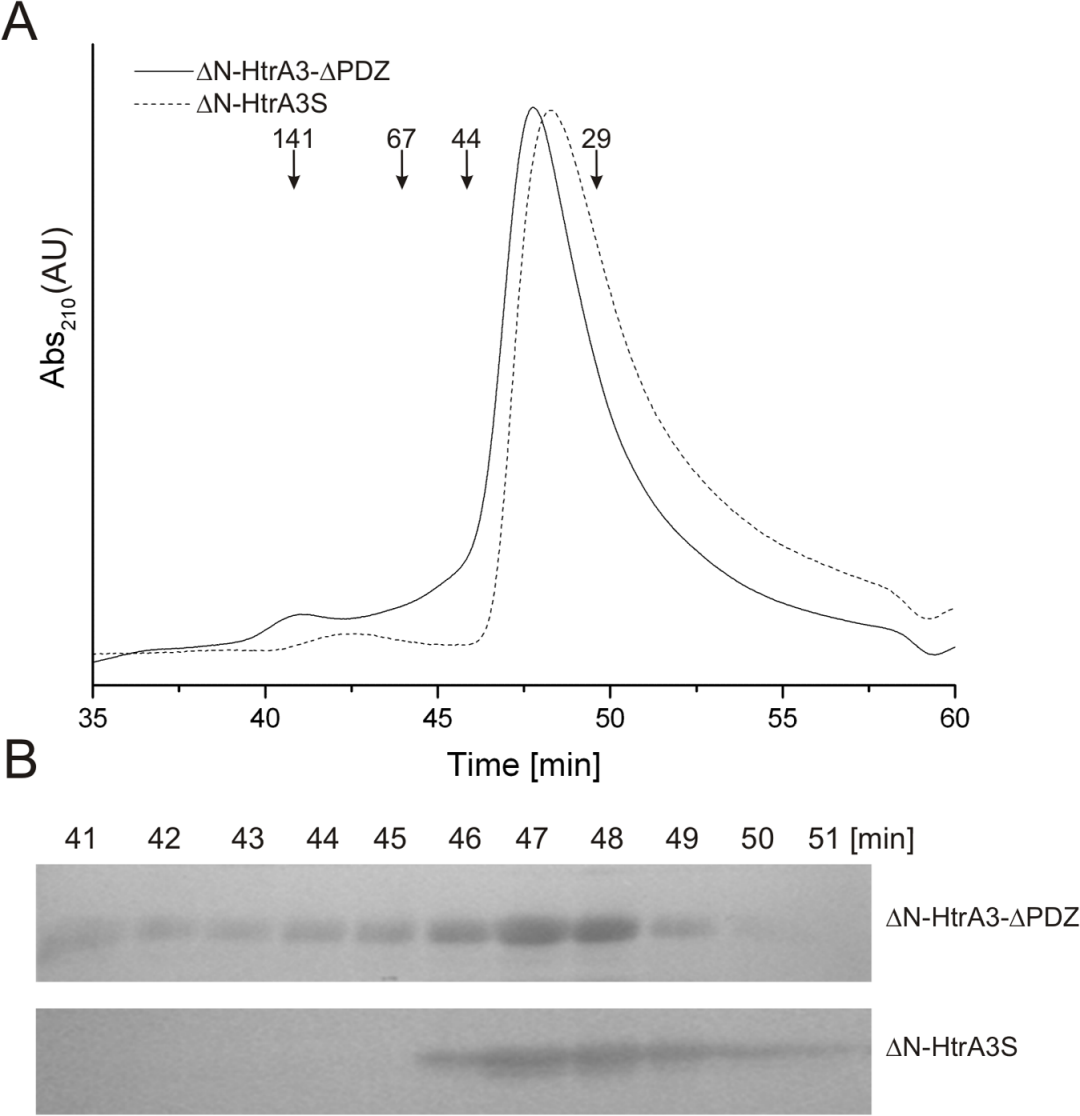
Influence of the PDZ domain on ΔN-HtrA3 oligomerization. (A) The ΔN-HtrA3-ΔPDZ and ΔN-HtrA3S proteins (50 μl at 3 mg/ml) were fractionated using size exclusion chromatography. Positions of molecular weight standards are marked with arrows. (B) The ΔN-HtrA3-ΔPDZ and ΔN-HtrA3S protein fractions were analyzed with SDS-PAGE followed by silver staining.

SEC of ΔN-HtrA3-ΔPDZ and the ΔN-HtrA3S isoform was performed to determine whether the PDZ domain influences HtrA3 oligomerization. Both variants were proteolytically inactive (i.e., with S305A substitution) to ensure homogeneity of the preparations. ΔN-HtrA3-ΔPDZ and ΔN-HtrA3S eluted as proteins with the apparent molecular mass of ~33 and ~35 kDa, respectively (Fig 10 and Table 4), which indicated that they were monomeric in solution. Since it was possible that these proteins formed trimers which were very unstable and dissociated during SEC, we performed crosslinking of the HtrA3 protein variants with Bis(sulfosuccinimidyl) suberate (BS^3^) to stabilize the oligomeric forms. Polyacrylamide gel electrophoresis (SDS-PAGE) of the crosslinked proteins confirmed that ΔN-HtrA3-ΔPDZ and ΔN-HtrA3S, in contrast to ΔN-HtrA3, did not form defined oligomers (data not shown). Hence, our results suggest that the presence of the PDZ domain is necessary to maintain the ΔN-HtrA3 trimeric structure. Also, it seems that the 7 C-terminal residues of HtrA3S do not influence protease oligomerization.

### Examination of cleavage specificity

**Fig 11:**
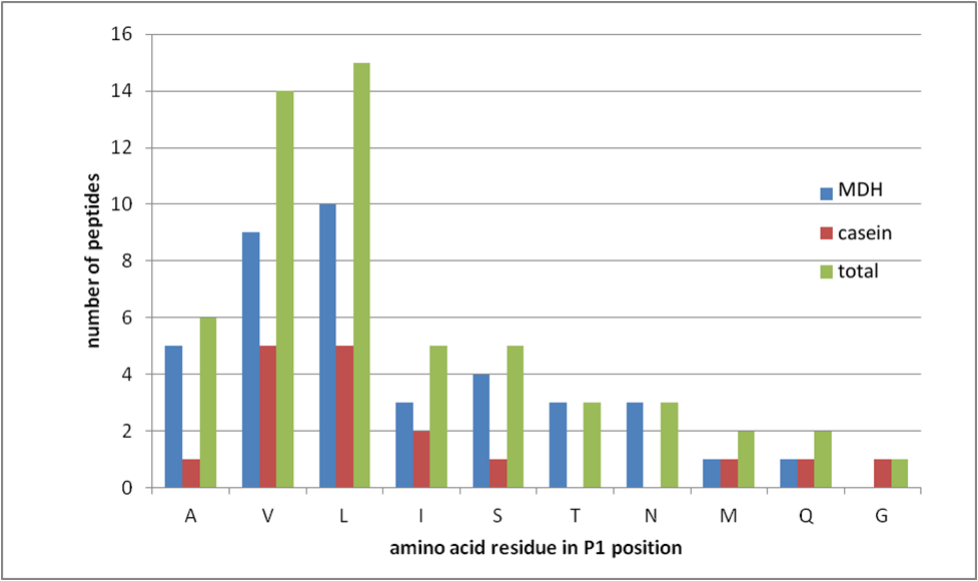
The primary specificity of ΔN-HtrA3 based on digestion products of malic dehydrogenase and bovine casein. P1 occurrence was plotted against the 10 amino acid residues that were identified in this position.

We evaluated ΔN-HtrA3 activity against a panel of potential model protein substrates (β-casein, malic dehydrogenase, citrate synthase, reduced lysozyme, bovine serum albumin, deoxyribonuclease I, ribonuclease A, carbonic anhydrase, cytochrome c, hemoglobin, α-amylase and goat immunoglobulin G) by analyzing the proteolytic products using SDS-PAGE and found appreciable activity only against β-casein and unfolded malic dehydrogenase. Activity against unfolded citrate synthase was detectable but lower. Since ΔN-HtrA3 has autoproteolytic activity (results not shown), we digested β-casein and malic dehydrogenase under conditions which allow a minimal ratio of the ΔN-HtrA3 to substrate proteolysis products to be obtained. To explore the cleavage specificity of ©N-HtrA3, we analyzed the initial products of the digests via mass spectrometry LC-MS. The identified cleavage sites are summarized in Table 6 and the frequency of amino acids occurring at the P1 position of substrates is shown in Fig 11. Based on the degradation products of both proteins we can assume that ΔN-HtrA3 is able to hydrolyze the peptide bonds with aliphatic or hydrophilic uncharged amino acid residues at position P1 (immediately amino-terminal to the cleaved peptide bond), with the prevalence of Leu and Val (followed by Ala, Ile, and Ser). The position P1 (carboxyl terminal to the cleavage site) also seems to accept aliphatic residues (Leu, Ala) and Ser; however, in some peptides the presence of Glu was observed. In position P2, there was a trend to accommodate the polar and charged residues, and Pro. In both P3 and P4, ΔN-HtrA3 preferred not only amino acids with aliphatic side chains (Leu or Ile) but also charged, Lys and Glu. P1 specificity is similar to that of HtrA2, which has strong preference for Val, Leu, Met, Ala, Ile, Gln, and Ser [37], and to that of HtrA1, with a preference for small- and medium-sized hydrophobic residues (Val, Leu, Met, Ala, and Ile) [9] [10]. ΔN-HtrA3 P1 specificity (i.e. Leu, Ala and Ser) resembles that of HtrA2, which prefers Ser, Ala and Thr [37]. In this position, HtrA1 accepts mainly polar uncharged residues (Thr, Tyr, Ser and Asn) [10]. At P2, the ΔN-HtrA3 preference (for polar and charged residues) partially differs from that of HtrA1 which accepts mainly aliphatic and charged amino acids. At P3, the ΔN-HtrA3 preference is similar to that of HtrA1 which accepts aliphatic (Leu, Val, Ala) and charged (Glu, Lys, Arg) residues. The P4 specificity of ΔN-HtrA3 partially resembles that of HtrA1, which prefers aliphatic residues; however, there is some difference, since ΔN-HtrA3, but not HtrA1, also accepts charged residues [10].

**Table 6:** xN-HtrA3 cleavage sites identified in0020-casein and malic dehydrogenase initial digestion products using LC-MS technique.

	P4	P3	P2	P1	P1'
**Bovine** β**–casein**	K	V	L	I	L
	L	S	S	S	S
	P	P	T	V	M
	L	E	E	L	N
	L	S	S	S	E
	P	L	P	L	L
	C	L	V	A	L
	L	E	E	L	N
	N	V	P	G	E
	L	S	S	S	E
	P	P	Q	S	V
	S	K	V	L	P
	Q	P	E	V	M
	E	L	N	V	P
	P	G	E	I	V
	V	M	G	V	S
	V	L	P	V	P
	L	P	P	L	Q
	Q	R	D	M	P
**Malic dehydrogenase**	H	G	S	L	F
	I	H	P	S	S
	G	V	N	V	A
	I	S	I	L	G
	S	V	I	N	Q
	E	D	K	L	G
	K	W	I	I	S
	D	S	S	V	A
	K	E	K	L	I
	V	I	K	L	K
	T	D	E	L	A
	A	I	S	I	L
	A	E	K	L	A
	K	I	V	A	D
	I	I	V	V	S
	D	E	L	A	L
	A	D	T	L	W
	K	M	V	V	E
	R	I	P	N	N
	L	N	L	V	Q
	V	V	T	A	G
	K	G	Y	T	N
	V	S	L	Q	E
	K	E	K	L	I
	K	G	E	M	M
	I	A	P	V	A
	V	V	T	A	S

Overlapping fragments with different length were omitted.

## Discussion

HtrA3 is a protease involved in the promotion of apoptosis and embryo implantation [20] [22] [23] [25] and linked to human diseases such as cancer and preeclampsia [24] [26] [27] [28] [29] [30] [31] [32]. We sought structural and biochemical insights into its function, so that its role in physiological conditions and disease may be better understood.

To date, only the structure of the PDZ domain of HtrA3 has been determined by X-ray crystallography [12]. We solved the crystal structure of the ΔN-HtrA3_S305A_ proteolytically inactive variant, encompassing the PD and PDZ domain (Figs 1, 2 and 3), and found that it is a homotrimer closely resembling the structures of two other human HtrA proteins: HtrA1 and HtrA2 [9] [11] [12]. The resolution of the HtrA3 structure, 3.27 Å (Table 2), is significantly lower than the average for protein structures deposited in the Protein Data Bank (PDB). However, the quality of diffraction was significant and allowed well-defined electron density maps and acceptable structure refinement statistics to be obtained (Table 3). Similar diffraction resolutions in the range 2.5–3.2 Å were obtained for HtrA1 structures [9]. The ΔN-HtrA3 trimer has a conical shape with peripheral PDZ domains pointing away from the center of the trimer, similarly as in HtrA1 or HtrA2. However, the positioning of the PDZ domains with respect to the PDs is intermediate between the open, saucer-like form of HtrA1 [9] and the closed form of HtrA2, where the PDZ domain restricts access to the protease active site [11].

The main structural difference in ΔN-HtrA3 PD compared to the domain structures of HtrA1 and HtrA2 is the LB loop (residues 189–203), which is six residues longer than their counterparts. The loop forms several hydrogen bonds with the PDZ domain, including five bonds in the 3–3.2 Å bond-distance range (Fig 6). Although the strength of bonding is substantial, it did not prevent disruption of the LB loop-PDZ interaction in chains B and C of the crystal structure. In our opinion, the ΔN-HtrA3 molecule represented by chain A shows a kinetically stable form of the protein monomer which may transform into either the open or closed forms reported for the HtrA1 and HtrA2 proteins, respectively. The open form of HtrA1 is present in either the active or inactive conformation [9], while the HtrA2 closed form is not accessible to a substrate [11]. The interaction between the xxN-HtrA3 PD and PDZ domains through the unique extended LB loop, which carries the catalytic H191 residue, may be involved in HtrA3 activity regulation. The interaction is stronger than that reported for HtrA2, where only van der Waals contacts were observed between the PD and PDZ domains [11]. Moreover, we do not exclude the possibility of forming a closed form of the ΔN-HtrA3 protein through van der Waals contacts between residues 321–330 of the HtrA3 PD and residues 360–372 of the PDZ domain, analogous to HtrA2. The postulated conformational transitions might coincide with changes in the activity.

The ΔN-HtrA3_S305A_ active site is virtually the same as that observed in HtrA1_S328A_ [9], with the catalytic serine residue (Ser305A) being too distant from His191 for proton transfer. Furthermore, the side-chain of L322 blocks access to the S1 specificity pocket similarly as L345 in the HtrA1 inactive PD (i.e., HtrA1_Cat(S328A) chain X [10]) (Fig 5B). Thus, our structure represents the inactive, resting conformation of the ΔN-HtrA3 protease. For other HtrA proteases it has been reported that the resting and active conformations differ in the arrangement of their regulatory and active site loops (LA, L1, L2, L3, LD) (reviewed by [19] [59]). The fact that parts of the LA and L3 loops (Fig 3) were not visible in the ΔN-HtrA3 structure suggests their mobility and thus the possibility that they change conformation upon substrate binding. Similarly, the L3 loop is not visible in the HtrA1 and the *E*. *coli* HtrA(DegP) inactive structures, but is present in the active conformations [9] [56]. The structure of the HtrA3 active form remains to be solved.

The basic unit of HtrA proteases is a trimer; however, some HtrAs may form higher oligomeric forms (6-, 12-, or 24-mers) without a substrate or upon substrate binding, and the change in oligomerization is usually correlated with stimulation of activity (reviewed by [2] [19]). Recently, using SEC, Truebestein et al. [9] found that human HtrA1 forms trimers that assemble into large multimers of approximately 600 kDa in the presence of an unfolded substrate, citrate synthase. Unfortunately, we were unable to visualize such well defined large multimers using several experimental approaches (SEC, crosslinking, analytical ultracentrifugation) and in the presence of various protein (β-casein, malic dehydrogenase and citrate synthase) or peptide (β-casein hydrolysate and the IRRVSYSF peptide) substrates. Our results do not exclude the possibility that HtrA3 may form high order oligomers, similarly as HtrA(DegP) or DegQ (reviewed by [19]), however they may be less stable. On the other hand, the trimers of *E*. *coli* DegS [5] and possibly HtrA2 of *Mycobacterium tuberculosis* [8] do not form high order oligomers.

ΔN-HtrA3 protease is similar to HtrA1 and HtrA2 not only structurally but also with respect to cleavage specificity, and preferentially cleaves peptide bonds formed by the carboxyl groups of hydrophobic, aliphatic amino acids (Table 6 and Fig 11). Its activity is highly stimulated by temperature (Fig 8), as shown previously for human HtrA2 [37] [38]. This result is in agreement with a report showing that HtrA3 stability is temperature dependent, due to autoproteolysis [35]. The observed features are typical for HtrA proteases, recognizing hydrophobic stretches of aberrant proteins arising under conditions of stress. Since ΔN-HtrA3 activity with model substrates is similar to that of HtrA2 ([38] and data not shown) but significantly lower than HtrA1 [10], there may exist an as yet unknown positive regulator of its activity.

The earlier reports showed that HtrA3L and HtrA3S proteins had similar levels of protease activity when assayed with β-casein, suggesting that the PDZ domain is not required for activity [35] [58]. In this study, using ΔN-HtrA3-ΔPDZ and ΔN-HtrA3S, we demonstrated that the PDZ domain has no apparent effect on enzymatic activity at a wide range of temperatures; furthermore it does not have significant influence on the substrate affinity of the protease (Figs 8 and 9). HtrA3 resembles HtrA1 in this respect, since it has been shown that the PDZ domain of HtrA1 is dispensable for activation [9]. The similarity between HtrA1 and HtrA3 is also highlighted by the fact that neither HtrA3 (this study and [34]) nor HtrA1 [10] require their N-terminal domains for protease activity. At the same time, HtrA3 differs from other characterized HtrAs, including human HtrA2, for which the PDZ domains are important for activity modulation. In the latter, the PDZ domain restricts substrate access to the active center and PDZ removal causes activity to increase [11]. Though the PDZ domain has no influence on ΔN-HtrA3 intrinsic activity assayed *in vitro*, it may bind specific, so far unknown, ligands in the cell and have a regulatory function. This is in agreement with the finding that exogenous full-length HtrA3L is more efficient than HtrA3-ΔPDZ in promoting the apoptosis of cancer cells [25].

Removing the PDZ domain transformed ΔN-HtrA3 into monomers and, furthermore, ΔN-HtrA3S was also monomeric in solution (Fig 10). This was unexpected, since the HtrA2 [11] and HtrA1 [9] proteins without PDZ and N-terminal domains (i.e. equivalent to the ΔN-HtrA3-ΔPDZ) are trimeric. The ΔN-HtrA3 structure does not reveal a clear reason for the monomerization of HtrA3 proteins lacking the PDZ domain. The PDZ domains in the reconstructed trimer structure are distant from the trimerization interface. Also, they do not interact with each other and the only contacts between the PDZ domain and the PD are via the LB loop. However, there is a significant difference in the free energies of the trimer dissociation for ΔN-HtrA1 and ΔN-HtrA3 proteins equal to 22.6 and 14.3 kcal/mol, respectively (calculated by PISA server-Protein Interfaces, Surfaces and Assemblies service (PISA) at European Bioinformatics Institute) [60]. Also, the buried area of the ΔN-HtrA3 trimer is 5630 Å^2^, which is significantly smaller than that of ΔN-HtrA1 (8810 Å^2^). This indicates that the HtrA3 trimer is less stable and could be disrupted more easily compared to HtrA1. It is possible that the presence of the PDZ domain, which closely interacts with the LB loop of the PD, stabilizes the structure of the PD and thus indirectly improves the stability of the trimer. In intact HtrA3L its N-terminal domains may influence trimer stability, especially if they are placed in the central core of the trimer, adjacent to the PDs. Recently such localization of the N-terminal domains has been proposed for HtrA1 [10].

Since the ΔN-HtrA3 variants without the PDZ domain are monomeric (Fig 10) and fully active (Figs 8 and 9), the trimer formation does not seem to be required for the enzymatic activity of HtrA3. This is in contrast to the situation observed in the case of HtrA2, which is inactive in its monomeric form [11]. On the other hand, the N-terminally truncated monomeric forms of the *E*. *coli* HtrA(DegP) are active; and, furthermore, such forms are present *in vivo* [61].

The N-terminally truncated HtrA3-ΔPDZ and HtrA3S have similar proteolytic activity (Figs 8 and 9) and are both monomeric (Fig 10) which suggests that the 7 C-terminal residues unique for HrA3S have no significant impact on the activity and oligomerization of ΔN-HtrA3S. Since HtrA3 is structurally similar to HtrA1 and the HtrA1 N-terminal domain appears to have no contact with the PDZ domain [10], it is probable that this also applies to the intact HtrA3S. The role of these C-terminal residues remains puzzling and should be further investigated.

In conclusion, our studies showed that although the structure of HtrA3 is similar to that of other human HtrAs, there are significant differences. The HtrA3 PDZ domain is dispensable for activity, as in HtrA1, but does not have an inhibitory effect, as observed in HtrA2. As determined by SEC, ΔN-HtrA3-ΔPDZ is monomeric, suggesting that, unlike in HtrA1 or HtrA2, the presence of the PDZ domain influences the formation of the HtrA3 trimer. Also, we were unable to demonstrate the formation of large multimers in the presence of a substrate, similar to those described for HtrA1 [9]. The PD structure of HtrA3, with the exception of the LB loop is strikingly similar to that of HtrA1. The LB loop may contribute significantly to the protein conformational flexibility as well as to the protein activity. Thus, HtrA3 seems to have a unique combination of features among human HtrA proteases.
